# In Vitro Corrosion Study of Friction Stir Processed WE43 Magnesium Alloy in a Simulated Body Fluid

**DOI:** 10.3390/ma9070542

**Published:** 2016-07-07

**Authors:** Genghua Cao, Datong Zhang, Weiwen Zhang, Wen Zhang

**Affiliations:** National Engineering Research Center of Near-Net Shape Forming for Metallic Materials, South China University of Technology, Guangzhou 510640, Guangdong, China; cghcaogenghua@126.com (G.C.); mewzhang@scut.edu.cn (W.Z.); jack_eei@scut.edu.cn (W.Z.)

**Keywords:** WE43 magnesium alloy, friction stir processing, corrosion behavior, mechanical properties

## Abstract

Corrosion behavior of friction stir processing (FSP) WE43 alloy in a simulated body fluid (SBF) was investigated. Micro-galvanic corrosion was the dominated corrosion behavior, and the corrosion resistance of FSP WE43 alloy was improved compared to the cast counterpart. Furthermore, due to the fine-grained and homogeneous microstructure, uniform corrosion morphology was observed on FSP WE43 alloy. According to the tensile properties of specimens with different immersion time intervals, FSP WE43 alloy shows better performance to maintain the mechanical integrity in SBF as compared to the as-cast alloy.

## 1. Introduction

Magnesium (Mg) alloys have excellent biocompatibility, which are desirable for medical implant materials [1,2,3,4,5,6,7]. However, due to their fast corrosion rates in the physiological environment, the corrosion resistance of Mg alloys need be improved to maintain their mechanical integrity before the tissue has sufficiently healed [8,9,10]. Rare earth (RE) elements are usually used to improve the high-temperature strength and creep resistance of Mg alloys, and some RE elements can simultaneously improve the corrosion resistance of Mg alloys. The addition of Y and Nd elements can enhance the tensile strength of Mg alloys, and also favor the formation of a protective surface layer, decreasing their corrosion rate [11,12,13,14,15]. Moreover, WE43 alloy has been successfully used in biomedical applications [16]. Therefore, it is expected that Mg-Y-Nd alloys will be widely used as biomedical implants.

Until present, the relationship between the microstructure and corrosion behavior of Mg alloys has not been fully understood [17,18]. Due to the different electrode potential between α-Mg matrix (anode) and secondary phases (cathodes), Mg alloys are highly susceptible to galvanic corrosion, so localized corrosion is often observed in Mg alloys, resulting in the decreased corrosion resistance of Mg alloys. For WE43 alloy, the corrosion pits are related to the galvanic coupling between α-Mg matrix and intermetallic compounds or impurities [19,20,21]. In some research, microstructure refinement is demonstrated to be one of the strategies to avoid seriously localized corrosion for Mg alloys [22,23]. For instance, Gu et al. reported that the Mg-3Ca alloy prepared by rapid solidification (RS) exhibited dramatically reduced degradation rate compared to the as-cast one, and the more uniform corrosion morphology was shown on the surface of RS specimen [22]. It is well known that ultrafine or nanocrystalline Mg alloys can be obtained by severe plastic deformation (SPD) techniques, such as high pressure torsion (HPT) and equal channel angular pressing (ECAP) [18,23,24]. However, both increased and decreased corrosion resistances are reported in SPD Mg alloys. Song et al. reported that high dislocation density and more energetic crystalline defects were introduced by SPD, these may increase the corrosion rate of pure Mg and AZ91D in NaCl solutions [25,26]. Therefore, the corrosion resistance of fine-grained Mg alloys need to be examined carefully, and special attention should be paid to the distribution of second-phase particles or microstructure defects.

As a novel SPD technique for microstructure modification, friction stir processing (FSP) has been demonstrated effective in fabricating fine-grained and homogeneous microstructure of metallic materials [27,28]. Compared to the extensive studies on mechanical properties, research on the corrosion behaviors of FSP materials is still limited. Ni et al. reported that the corrosion resistance of FSP NiAl bronze was significantly increased compared to the cast counterpart [29]. Argade et al. prepared different grain sizes of WE43 alloy by multi-pass FSP, and found that the corrosion rates in 3.5 wt % NaCl solutions decreased with the decreasing grain size [30]. After aging treatment, different corrosion behavior was observed under electrochemical and constant immersion tests for FSP WE43 alloy [31]. However, to the best of the authors’ knowledge, the corrosion behavior of FSP WE43 alloy in a simulated body fluid (SBF) has not been investigated until present. It is reported that the corrosion rate of WE43 alloy in SBF was significantly higher than that in simple NaCl solutions [32]. Since the ion concentrations in SBF are nearly equal to those of human blood plasma, the corrosion behavior in SBF is helpful to predict the degree of in vivo bone bioactivity of the material [33]. For the purpose of being a biomedical implant, it is necessary to investigate the corrosion behavior of FSP WE43 alloy in SBF. In this study, WE43 alloy with fine-grained and homogeneous microstructure was prepared by FSP, the corrosion behavior of specimens in SBF was investigated, and the aim focuses on the influence of FSP on corrosion mechanism of WE43 alloy.

## 2. Results

### 2.1. Microstructure

**Figure 1:**
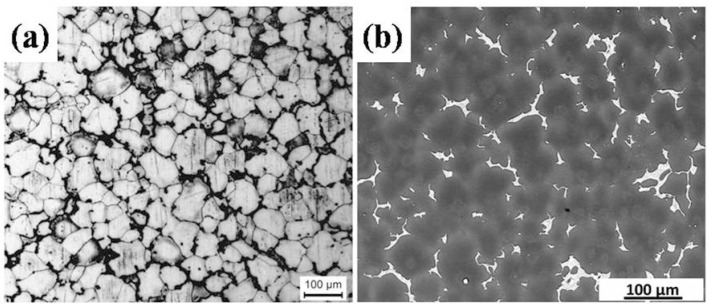
(**a**) OM and (**b**) BSE images of the BM specimen.

Figure 1 shows the microstructure of the base material (BM) specimen. As shown in Figure 1a, the average size of coarse α-Mg grains is ~53 μm measured by the mean linear intercept method. Figure 1b shows the back-scattered electron (BSE) image of second phases distributed randomly in the BM. According to our previous work [34], the main coarse second phases in the BM are Mg_12_Nd (coarse eutectic networks mainly located at the grain boundaries) and Mg_24_Y_5_ (cubic-shaped particles mainly decorated in the intragranular region), respectively.

**Figure 2:**
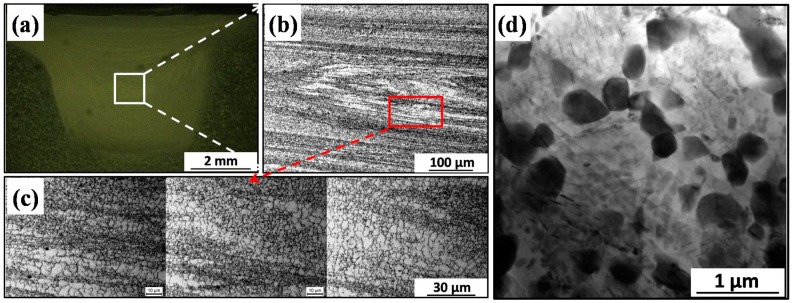
(**a**) Cross-section macrograph of the FSP specimen; (**b**,**c**) OM image of the center region in the SZ; and (**d**) TEM image showing second phase particles in SZ of FSP specimen.

Figure 2 shows the microstructure of WE43 alloy after FSP. The cross-section macrograph of FSP specimen is shown in Figure 2a, no obvious defects can be found in the stirred zone (SZ), indicating the process is performed successfully. The microstructure in the central region of the SZ are shown in Figure 2b, in comparison with the BM specimen, the coarse grains are significantly refined after FSP, which is mainly attributed to the dynamic recrystallization (DRX) behavior during FSP [28]. The average grain size of FSP specimen is calculated as ~2.7 μm (Figure 2c). Furthermore, due to the extensive plastic deformation during FSP [28], the coarse second phases in the BM are broken into fine particles in the SZ (Figure 2d).

### 2.2. Corrosion Behavior

#### 2.2.1. Electrochemical Corrosion Test 

**Figure 3:**
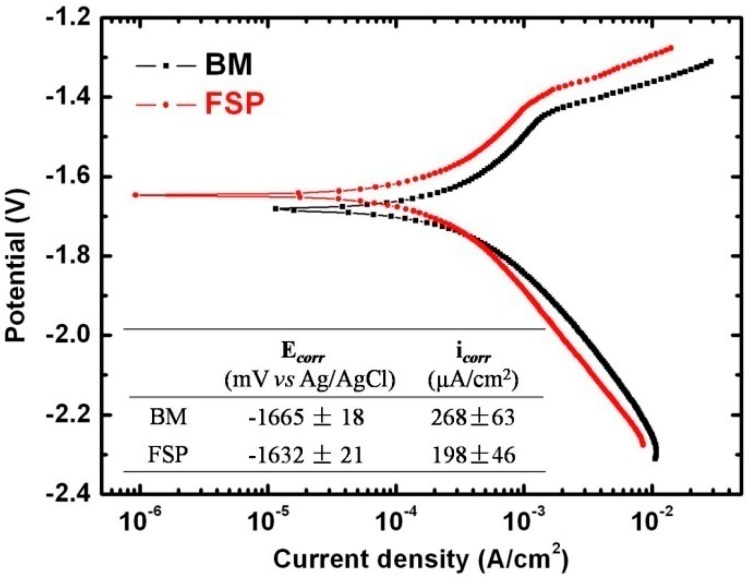
Potentiodynamic polarization curves of the BM and FSP specimens in SBF.

The electrochemical polarization behavior of the specimens in SBF at 37 °C is illustrated in Figure 3. According to the similar polarization curves, there is no significant difference in the polarization behavior between the BM and FSP. The electrochemical data are evaluated by Tafel extrapolation from polarization curves. As shown the inserted table in Figure 3, the corrosion potential (E*_corr_*) of the FSP specimen (−1632 ± 21 mV vs. Ag/AgCl) is more positive than that of BM specimen (−1665 ± 18 mV vs. Ag/AgCl), the corrosion current density (i*_corr_*) of the FSP specimen (198 ± 46 μA/cm^2^) is slightly lower that of the BM specimen (268 ± 63 μA/cm^2^). Both of the E*_corr_* and i*_corr_* values indicate that the corrosion resistance of WE43 alloy is improved after FSP. Similar results are shown in [30], in which the electrolyte is 3.5 wt % NaCl solutions.

#### 2.2.2. Immersion Corrosion Test

**Figure 4:**
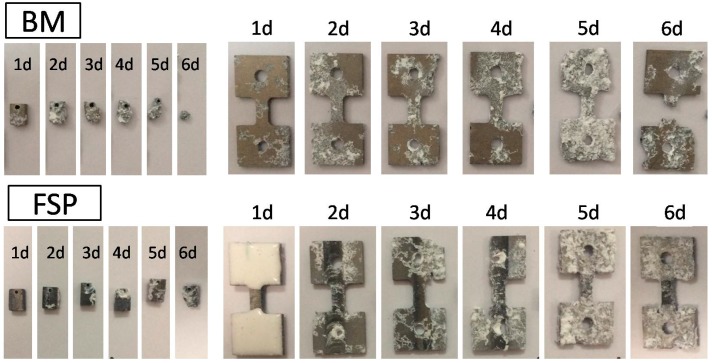
Typical overview surface appearance of specimens with different immersion time intervals in SBF (with corrosion products).

Figure 4 presents the typical overview surface appearances of the BM and FSP specimens after the immersion corrosion test in SBF. In order to observe the whole corrosion morphologies of the tensile test specimens after immersion, the epoxy resin covered on the grip region are removed. Due to the poor corrosion resistance, the maximum immersion time of BM is six days, in which the gage region of the tensile specimen and the weight loss specimen are almost dissolved completely in SBF. However, most areas of the FSP specimens are maintained at the same immersion time interval. This indicates that the corrosion resistance of FSP specimen is better than the BM specimen.

**Figure 5:**
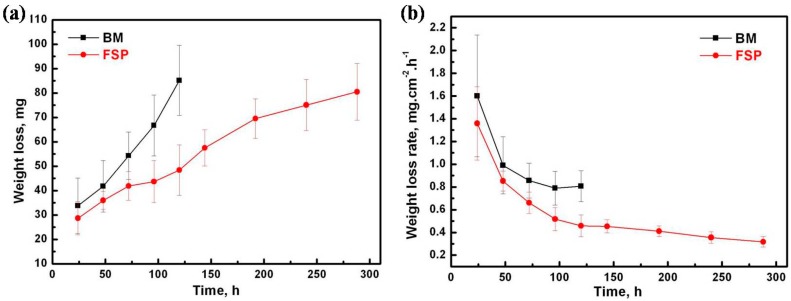
(**a**) The weight loss and (**b**) corrosion rate curves of specimens during immersion test.

The weight loss and corrosion rate curves of the specimens during immersion tests are shown in Figure 5. The corrosion rates of the specimens are calculated as Equation (1) [10,35]:

*CR* = *W*/*Atρ* (1)

where *CR* refers to the corrosion rate; *W* is the measured weight loss of the specimen; *A* is the exposure area; *t* is the immersion time; *ρ* is the standard density. For BM specimens, the weight loss significantly increases with increasing test time, and almost completely dissolved in SBF at six days. The corrosion rate of BM specimens is calculated to be 38.41 mm/yr. The weight loss for the FSP specimen is much lower compared to the BM specimen, and the corrosion rate is calculated to be 15.12 mm/yr. It demonstrates that FSP can effectively decrease the corrosion rate of as-cast WE43 alloy.

#### 2.2.3. Corrosion Morphology 

**Figure 6:**
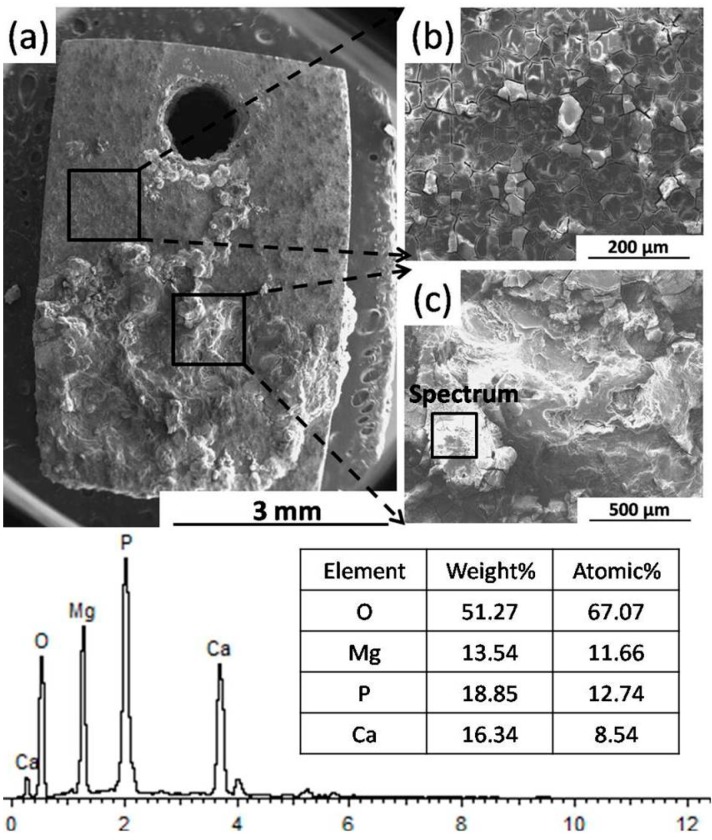
Surface morphology of the BM specimen (at different magnification) after immersion for one day, and the corresponding EDS spot analysis. (With corrosion products).

Figure 6a shows the serious localized corrosion morphology of the BM specimen after immersion test for one day. Both uniform protective film and thick corrosion film can be seen in Figure 6b,c, respectively. The main corrosion products of uniform film are reported to be Mg(OH)_2_ or perhaps MgO [36,37,38], and the cracks may be due to the dehydration reaction occurring during the drying process. The thick corrosion film correlates with a more serious corrosion behavior, indicating a faster corrosion rate [38]. EDS spot analysis shows that the main corrosion products of thick film are calcium phosphate and oxides of Mg.

**Figure 7:**
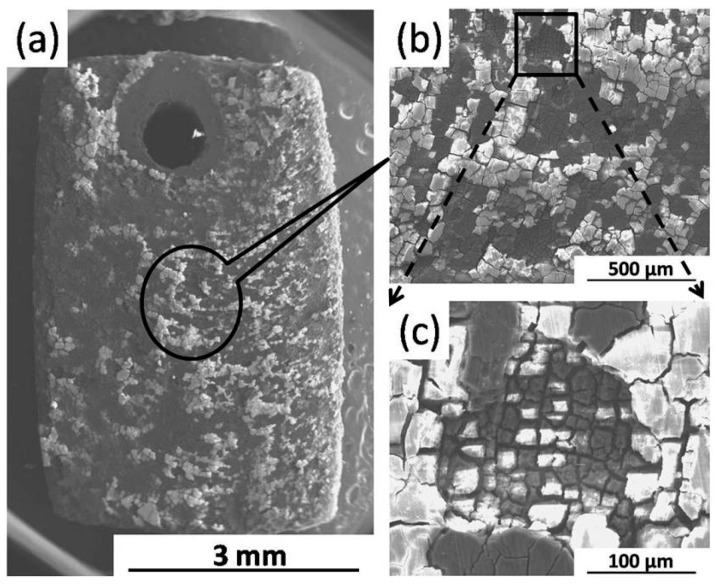
Surface morphology of FSP specimen (at different magnification) after immersion for one day (with corrosion products).

Figure 7a shows the uniform corrosion morphology of the FSP specimen after immersion test for one day. As shown in Figure 7b, a small pit is located on the surface of film. At a higher magnification, a uniform film is observed at the bottom of the pit (Figure 7c). This means that a new uniform film can re-form when localized corrosion occurred during the immersion test for the FSP specimen.

**Figure 8:**
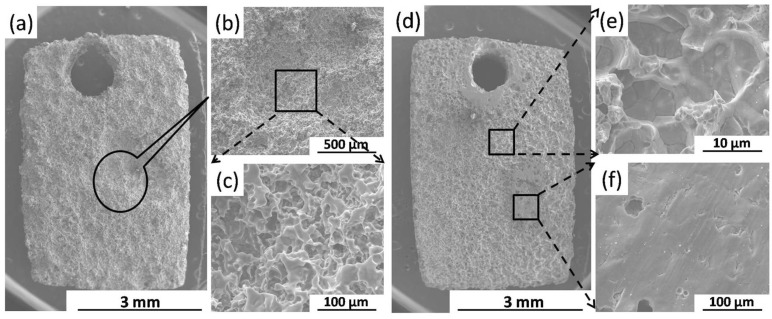
Surface morphologies of (**a**–**c**) the BM and (**d**–**f**) the FSP specimens after immersion for one day (without corrosion products).

After removing corrosion products, the surface morphologies of the BM and FSP specimens after immersion test for one day are presented in Figure 8. Figure 8a shows that the whole surface of the BM is seriously corroded, and the deeply corroded area is illustrated in Figure 8b. It can be found that plenty of deep pits are shown in this area (Figure 8c), which means that the localized corrosion penetrates deeply into the BM specimen. Figure 8d presents a smooth corroded surface of the FSP specimen. Although most of the surface areas are corroded after immersion for one day (Figure 8e), the shallow pits shown in these areas indicate that the localized corrosion do not penetrate deeply into the FSP specimen. In addition, there are still some original areas (Figure 8f), which suffered slight corrosion attack.

### 2.3. Tensile Test

#### 2.3.1. Tensile Properties

**Figure 9:**
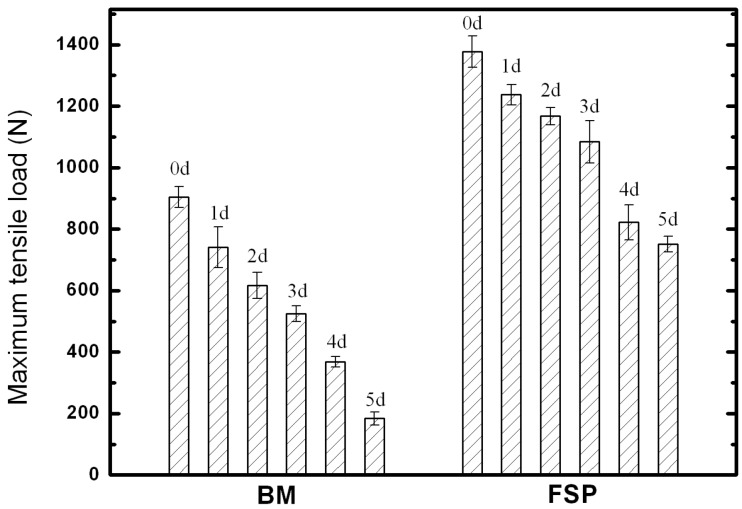
Tensile properties of the BM and FSP specimens after different immersion time intervals.

The results of tensile tests of BM and FSP specimens with different immersion time intervals are summarized in Figure 9. Since the load area of specimens is changed after immersion tests, only maximum tensile load (MTL) are provided for comparing. The original MTL of the BM and FSP specimens are 905 and 1378 N, corresponding to the ultimate tensile strengths of 199 and 303 MPa, respectively. After the immersion test for five days, the MTL of the BM specimen significantly decreases to 184 N, which is only 20% of the original value. Under the same test conditions, the MTL of the FSP specimen is 751 N, which is still 54% of the original value. Therefore, the FSP WE43 alloy shows better performance in maintaining the mechanical integrity in SBF as compared to the as-cast alloy.

#### 2.3.2. Fracture Morphology

**Figure 10:**
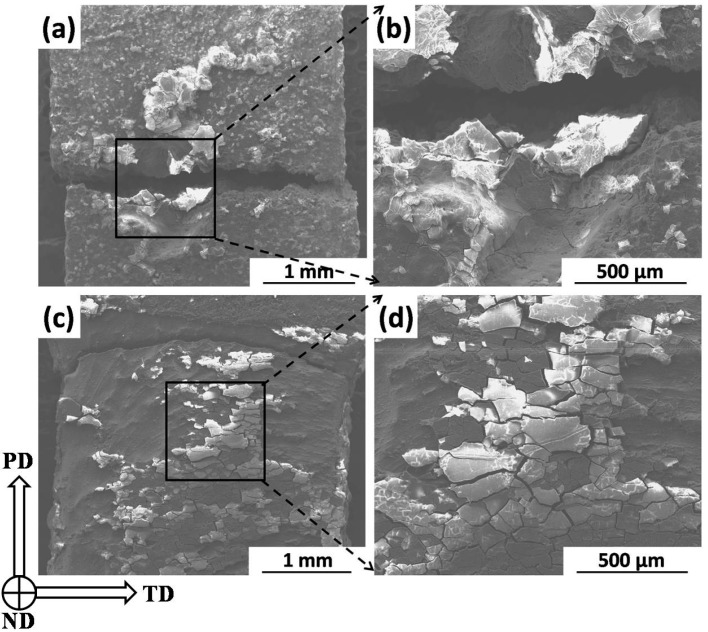
SEM images of tensile fracture appearance: (**a**,**b**) BM specimen; and (**c**,**d**) FSP specimen (after immersion for one day).

Figure 10 reveals the fracture appearances (observed along the normal direction (ND)) of the BM and FSP specimens after immersion for one day. Thick corrosion products are accumulated near the fracture of the BM specimen (Figure 10a), and a large corrosion pit is located at the fracture (Figure 10b). It can be concluded that the cracks are nucleated and propagated easily in these areas. For the FSP specimen, a relative smooth fracture surface is observed (Figure 10c). Some corrosion products are broken and separated during the tensile test, while some residual films are still attached on the fracture surface (Figure 10d).

**Figure 11:**
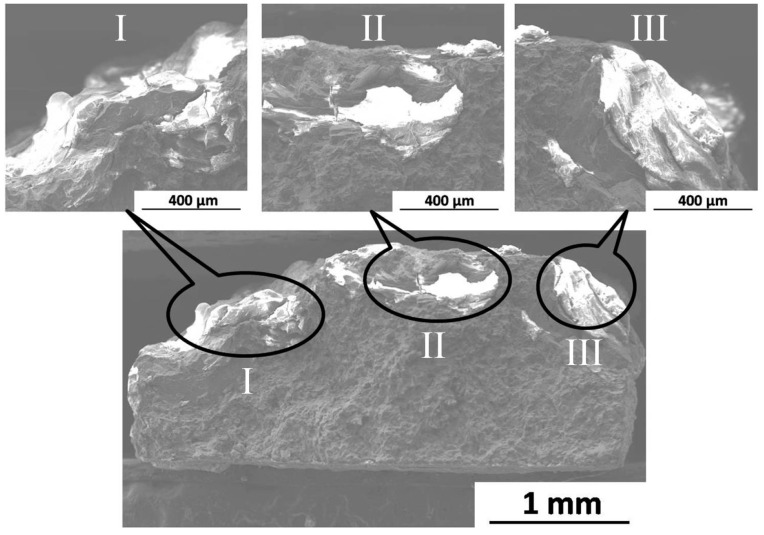
Fracture surface of the BM specimen after immersion for three days.

Figure 11 gives the fracture morphologies (observed along the PD) of the BM specimen after immersion for three days. It can be seen that plenty of material is dissolved into SBF during immersion test, and the irregular shape fracture surface is exhibited. In addition, a mass of white corrosion products are accumulated on the fracture surface, which have suffered serious corrosion as shown at locations I, II, and III in Figure 11. Therefore, the localized serious corrosion behavior is the main reason for the fracture of the immersed BM specimen.

**Figure 12:**
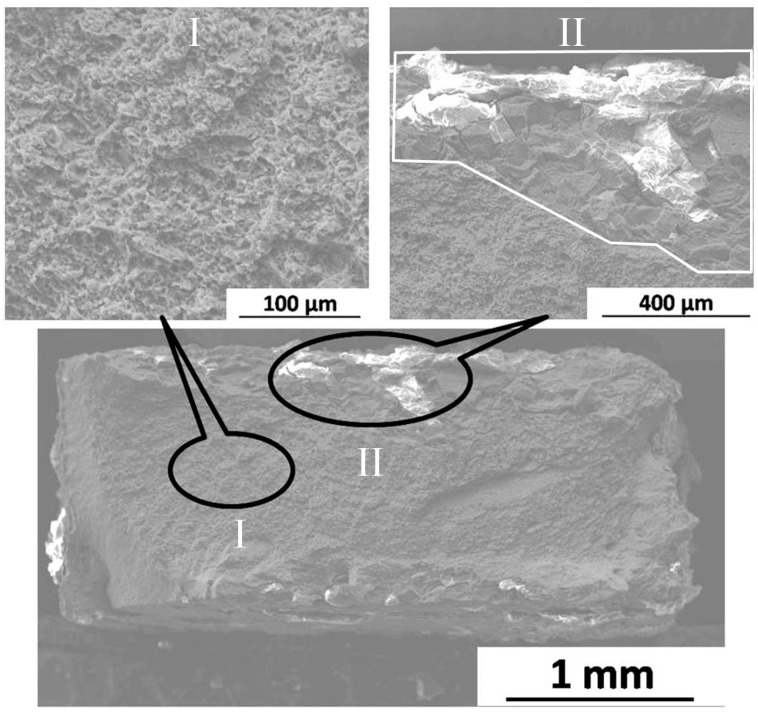
Fracture surface of FSP specimen after immersion for three days.

Figure 12 exhibits the TD fracture morphologies of FSP specimen after immersion for three days. The fracture surface almost maintains the rectangular shape, and dimples can be observed on the surface (location I in Figure 12), which is the typical characteristic of ductile fracture mode. However, a small quantity of corrosion products are examined on the side of fracture surface (marked in location II), and cleavage facets can be seen around corrosion products. It is speculated that the failure mechanism may transform into brittle fracture mode due to the corrosion attack. Compared to Figure 11, relatively uniform corrosion behavior can be observed on the FSP specimen.

## 3. Discussion

In recent years, a new class of biodegradable metals has attracted great attentions. These kinds of metals are expected to corrode gradually in vivo, and then dissolved completely upon fulfilling the mission to assist with tissue healing [10,39]. Mg and its alloys are promising candidates for the application of biodegradable metals, due to their mechanical and corrosion characteristics in the physiologic environment [8,40]. In order to predict the corrosion rate of biodegradable implants during their service period, uniform corrosion behavior of Mg alloys is desirable. In this study, the corrosion resistance and uniform corrosion ability of WE43 alloy in SBF are both significantly improved after FSP. In order to summarize the influence of FSP on corrosion behavior of WE43 alloy, the fine-grained structure and second phase particles are discussed, respectively.

### 3.1. Influence of Grain Refinement on Uniform Corrosion Behavior

Grain boundaries are high-energy areas in the microstructure, acting as an anode compared to the grain interior during galvanic corrosion [30]. Therefore, the fine-grained microstructure may be corroded easily due to the large volume fraction of grain boundaries per unit area. However, there is no inversely proportional relationship between grain size and corrosion rate, both increase and decrease corrosion resistances are shown with the grain refinement [18,23,24]. This is due to the physical or chemical properties of materials may be changed with the grain size modification by any processing and/or alloy addition, which also influence the corrosion rate of materials [18].

**Figure 13:**
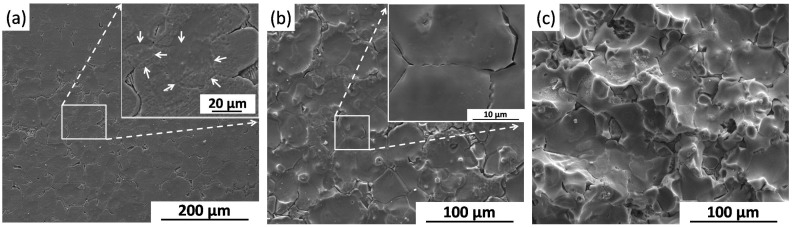
Surface morphologies of BM specimens after different immersion time intervals in SBF: (**a**) 0.5 h; (**b**) 1 h; and (**c**) 12 h (without corrosion products).

In the present study, the average grain size of BM is about 53 μm, and the surface morphologies of BM specimens with different immersion time intervals in SBF are shown in Figure 13. After immersion for 0.5 h, the areas with second phases are slightly corroded, while the grain boundaries without second phases cannot be observed clearly (as marked by white arrows in Figure 13a), indicating that the areas near the second phases are corrosion attacked prior to the grain boundaries. After immersion for 1 h, the localized corrosion morphology is shown in Figure 13b, and the grain boundaries are etched seriously. With the increasing of immersion time to 12 h, a much more serious corroded surface is exhibited (Figure 13c), and some grains are separated from the matrix and/or dissolved into SBF. It is presumable that when the grain boundaries are seriously corroded, the whole coarse grains with protective films may be undercut and separated, so that the further corrosion occurs in underneath microstructure, resulting in the poor corrosion resistance and the seriously-localized corrosion of BM specimen.

**Figure 14:**
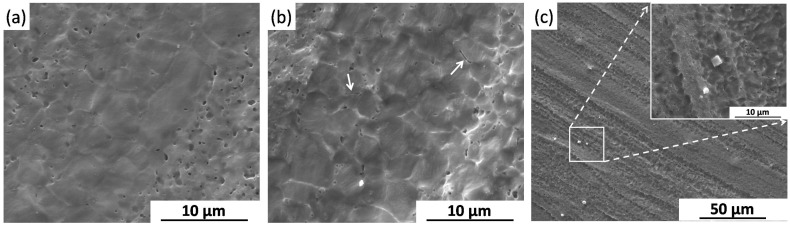
Surface morphologies of FSP specimens after different immersion time intervals in SBF: (**a**) 1 h; (**b**) 3 h; and (**c**) 12 h (without corrosion products).

After FSP, the α-Mg grains of BM are significantly refined to about 2.7 μm (Figure 2), and the surface morphologies of FSP specimens after different immersion time intervals in SBF are shown in Figure 14. After immersion for 1 h, the grain boundaries are ambiguous (Figure 14a), with the increasing immersion time to 3 h, the grain boundaries are slightly etched and the shape of grains can be observed (Figure 14b). It can be noticed that the grain boundaries of FSP specimens are harder to corrode than that of the BM specimens, which may be attributed to the enhancement of passivation kinetics of fine-grained microstructure [41]. In addition, the mismatch and disorder between the oxide layer and metal surface are both reduced for materials with higher grain boundary densities, leading to better adherence of protective films and, thus, it contributes to the decrease of corrosion rate [31,42]. This is one of the reasons for the formation of uniform protective film on the surface of FSP specimen (Figure 7). After immersion for 12 h, the fine grains are also separated from the matrix and/or dissolved into SBF (Figure 14c). Unlike BM specimen, when the finer grains with protective films fall off, the underneath areas for further corrosion are much smaller. Therefore, the better corrosion resistance and uniform corrosion behavior are obtained after FSP. This is also confirmed by electrochemical results shown in Figure 3.

### 3.2. Influence of Second Phases on Uniform Corrosion Behavior 

The electrical potential, volume fraction, size, and distribution of second phases have pronounced influences on the corrosion behavior of Mg alloys [2,17]. In our research, the main phases in the specimens are α-Mg matrix, Zr-rich particles, Mg_12_Nd, and Mg_24_Y_5_ [34,43]. Coy et al. reported that the Volta potential values of Mg_12_(Nd,Y) phase, Zr-rich, and Y-rich particles are all higher than the α-Mg matrix in wrought WE43-T6 alloy [44]. This means that the α-Mg matrix acts as an anode while the second phases act as a cathode when suffering galvanic corrosion in SBF, resulting in the heavy localized corrosion of α-Mg matrix adjacent to second phases. The micro-galvanic coupling between α-Mg matrix and second phases can be expressed as the following partial reactions [17]:

Mg → Mg^2+^ + 2e^−^ (anodic reaction) (2)

2H_2_O + 2e^−^ → H_2_ + 2OH^−^ (cathodic reaction) (3)

Mg^2+^ + 2OH^−^ → Mg(OH)_2_ (product formation) (4)

**Figure 15:**
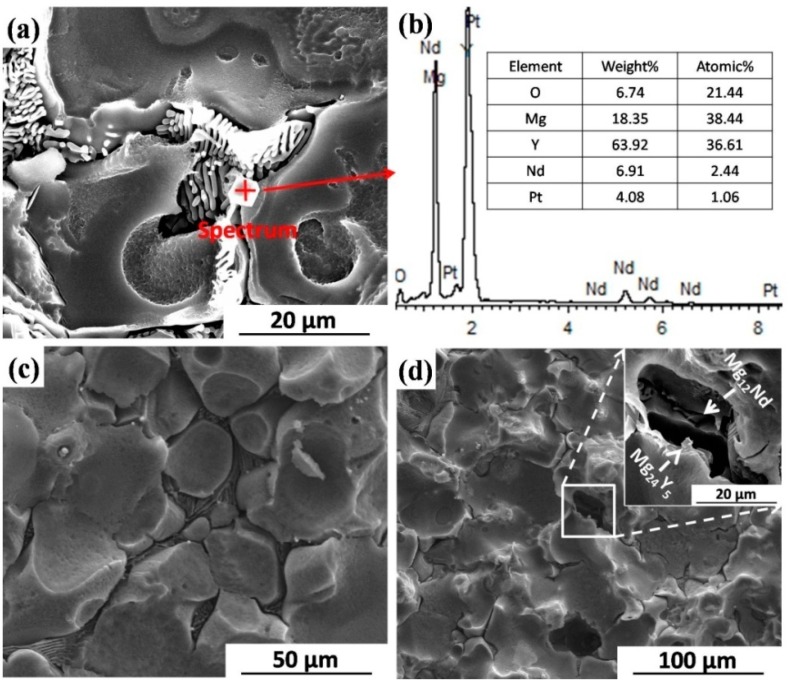
Surface morphologies of BM specimens after different immersion time intervals in SBF and the corresponding EDS spot analysis: (**a**,**b**) 3 h; (**c**) 6 h; and (**d**) 12 h (without corrosion products).

The influences of coarse second phases on corrosion behavior of BM specimens are shown in Figure 15. After immersion in SBF for 3 h, the grain interior adjacent to second phases is seriously corroded, while the Mg_12_Nd and Mg_24_Y_5_ phases are still decorated at grain boundaries (Figure 15a,b). After immersion in SBF for 6 h, the α-Mg matrix is further corroded (Figure 15c). After immersion in SBF for 12 h, the coarse second phases are undercut and fall out, leaving the deep holes so that the electrolyte can be penetrated into the underneath microstructure (Figure 15d). Song et al. found that if the β-phase is nearly continuous like a net over the fine α-Mg grains in die-cast AZ91D alloy, the β-phase particles do not easily fall out by undermining [45]. In this study, the grains in BM are coarse and the volume fraction of second phases is not high enough (Figure 1b), so the corrosion behavior is not effectively blocked by the second phases. In an even worse case, the separation of second phases leads to the much more seriously-localized corrosion in BM specimens.

**Figure 16:**
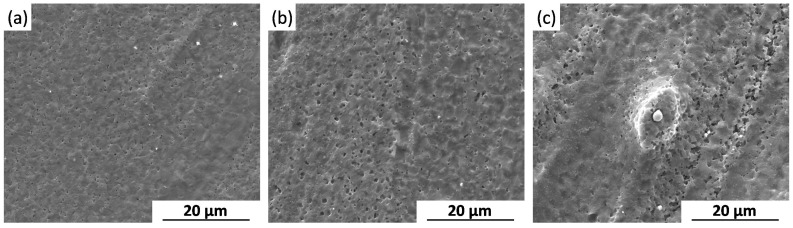
Surface morphologies of FSP specimens after different immersion time intervals in SBF: (**a**) 3 h; (**b**) 6 h; and (**c**) 12 h (without corrosion products).

After FSP, the coarse second phases are broken into fine particles (Figure 2d). The surface for the FSP specimen suffers serious micro-galvanic corrosion at the early stage of the immersion test (Figure 16a), and the corrosion is aggravated with the increased immersion time (Figure 16b,c). Even so, corrosion pits with smaller size and shallower depth are observed on the surface of FSP specimen. It means that the FSP specimen suffers slight corrosion attack compared to the as-cast one. Lunder et al. reported that the uniformly-distributed, fine second-phase particles are the most detrimental to the corrosion resistance of magnesium-base alloys [46]. However, Chu et al. reported that the finely-distributed precipitates in a peak-aged specimen decrease the corrosion rate of WE43 alloy [38]. The former one considered that the micro-galvanic couplings between particles and α-Mg matrix increased with the refined particles, while the later one thought that the precipitates played a prominent role in slowing down the corrosion reactions underneath. In the present study, micro-galvanic corrosion is the dominant corrosion mechanism for BM and FSP specimens. The refined microstructure and homogenous distribution of second phase particles in FSP alloy decrease the undercut effect during corrosion. This may be the main reason for the corrosion resistance is improved and the uniform corrosion morphology is exhibited in FSP WE43 alloy.

## 4. Materials and Methods

**Figure 17:**
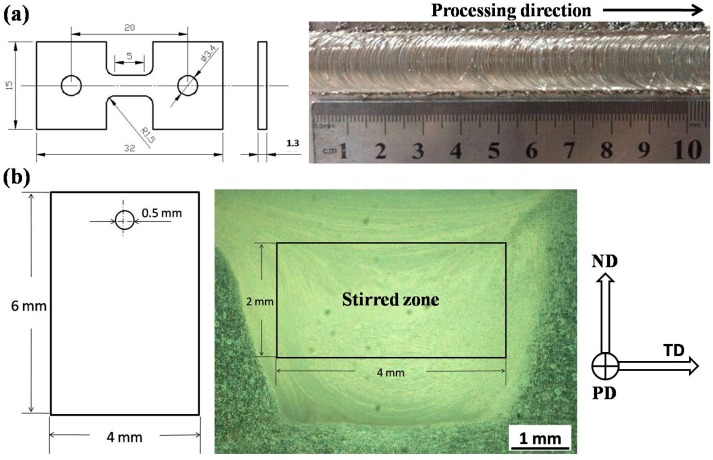
(**a**) Schematic diagram of tensile specimen and the surface appearance of processed region; and (**b**) schematic diagram of immersion specimen and the cross-section macrograph of the FSP specimen.

Cast Mg-Y-Nd alloy was used as BM in this study, and the chemical compositions are listed in Table 1. Plates with a thickness of 6 mm were cut from cast billets and then subjected to single-pass FSP at a rotation rate of 600 rpm and a traveling speed of 60 mm·min^–1^. A tool with a shoulder of 15 mm in diameter, a threaded conical pin of 4 mm in root diameter and 5 mm in length was used. The tool tilt angle was 2.5°. As shown in Figure 17a, tensile specimens with a gauge length of 5 mm, a thickness of 1.3 mm and a width of 3.5 mm were machined parallel to the processing direction (PD) with the gauge completely within the stirred zone (SZ). Immersion specimens with a size of 6 × 4 × 2 mm^3^ cuboids were also cut from SZ (Figure 17b).

Specimens for optical microscopy (OM) and scanning electron microscopy (SEM) observations were cut perpendicular to the processing direction, and prepared by mechanical polishing, then etching with a solution of 5 g picric acid +10 mL acetic acid +10 mL distilled water +80 mL ethanol. Thin foils for transmission electron microscopy (TEM) were ion–milled by a Gatan 691 miller at a voltage of 4 kV.

Potentiodynamic polarization was utilized to evaluate the corrosion behavior. The electrochemical measurements were conducted in SBF using an electrochemical workstation (IM6ex, Zahner, Germany). According to the preparation procedure of Ref. [33], the SBF used in this study containing 8.035 g/L NaCl, 0.355 g/L NaHCO_3_, 0.225 g/L KCl, 0.231 g/L K_2_HPO_4_·3H_2_O, 0.311 g/L MgCl_2_·6H_2_O, 0.292 g/L CaCl_2_, 0.072 g/L Na_2_SO_4_, and 6.118 g/L Tris (HOCH_2_)_3_CNH_2_. The experiments were performed in a three-electrode cell with a platinum electrode as the counter electrode, samples with an exposed area of 1 cm^2^ as the working electrode, a saturated Ag/AgCl electrode as the reference electrode. Specimens were immersed in SBF at 37 °C for 30 min to achieve constant potential before electrochemical experiments. The potentiodynamic polarization was measured at a scanning rate of 5 mV/s. Electrochemical experiments were conducted in triplicate to verify the validity of results.

**Figure 18:**
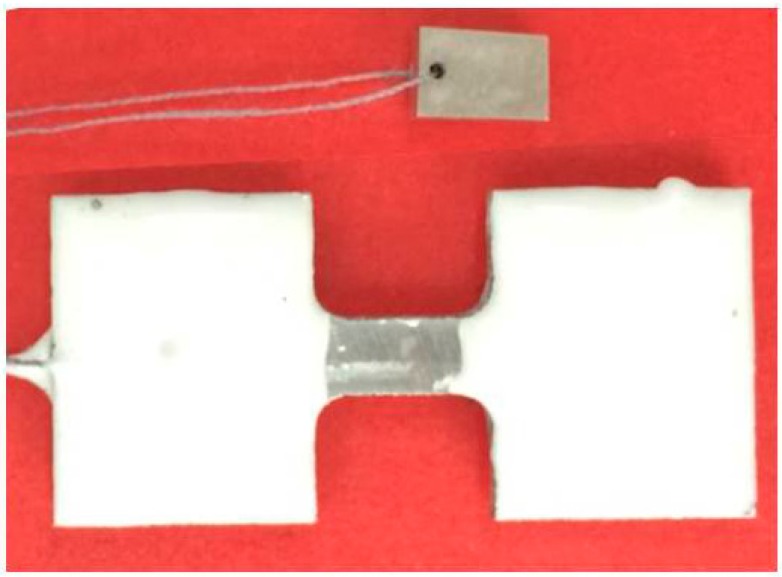
Image of specimen before immersion.

The immersion test was carried out according to ASTM-G31-72 in SBF and the temperature was kept at 37 °C by water bath. Figure 18 shows the weight loss specimen (small one) and tensile test specimen (big one), which are subjected to a multistage grinding process and immersed in SBF simultaneously. The weight loss specimens are used to measure the corrosion rate of FSP WE43 alloy in SBF, and the tensile test specimens are used to measure the variation of mechanical properties of FSP WE43 alloy with the different immersion time intervals. Furthermore, the grip region of tensile test specimens was covered with epoxy resin, which guaranteed only the gage region of the specimens was immersed in the SBF throughout the test procedure. In order to ensure the sufficient contact between specimens and solution, specimens were hung in the middle of SBF. During the corrosion tests, the electrolyte was changed every 48 h. The ratio of solution volume to specimen area was 0.31 mL/mm^2^ in this study. An average of three measurements was taken for each group.

After different immersion time intervals, the specimens were ultrasonically cleaned in distilled water, then immersed in 10 g/L CrO_3_ solution to clean the corrosion products and ultrasonically cleaned in distilled water. The surface morphologies of the specimens before and after removing corrosion products were characterized by SEM, equipped with an energy-dispersive spectrometer (EDS) attachment. Weight loss and corrosion rate (mm/yr) were measured. Tensile tests were performed on a SANS CMT5105 machine with a strain rate of 2 × 10^−3^ s^−1^. Tensile fracture morphologies of TD (transverse direction, transverse to the processing direction) and PD were subjected to SEM examination. The definition of the directions mentioned above is shown in Figure 17.

## 5. Conclusions

In this study, as-cast and FSP WE43 alloy were immersed in SBF for various time intervals to investigate their biodegradable behaviors. The following conclusions can be drawn:

1. The E*_corr_* and i*_corr_* values obtained by electrochemical measurements indicate that the corrosion resistance of as-cast WE43 alloy is improved after FSP. In addition, according to immersion tests, the corrosion rate of as-cast WE43 alloy is decreased from 38.41 mm/yr to 15.12 mm/yr after FSP.
2. Micro-galvanic corrosion is the dominant corrosion behavior for BM and FSP specimens. Due to the fine-grained and homogeneous microstructure, the uniform corrosion morphology was observed on FSP WE43 alloy.
3. After immersion in SBF for five days, the maximum tensile load for as-cast WE43 alloy is significantly decreased to 20% of the original value, while the maximum tensile load of the FSP specimen is still 54% of the original value, which is attributed to the improved corrosion resistance and uniform corrosion behavior after FSP.

## Figures and Tables

**Table 1:** Chemical compositions of as-cast Mg-Y-Nd alloy (mass fraction, %).

Mg	Y	Nd	Zr	Al	Ni	Si	Ca	Zn
Bal.	4.27	2.94	0.51	0.07	0.05	0.05	0.04	0.01
